# Graphical Evaluation of the Ridge-Type Robust Regression Estimators in Mixture Experiments

**DOI:** 10.1155/2014/806471

**Published:** 2014-08-18

**Authors:** Ali Erkoc, Esra Emiroglu, Kadri Ulas Akay

**Affiliations:** ^1^Department of Statistics, Faculty of Science and Letters, Mimar Sinan Fine Arts University, Sisli, 34380 Istanbul, Turkey; ^2^Department of Mathematics, Faculty of Science, Istanbul University, Beyazit, 34134 Istanbul, Turkey

## Abstract

In mixture experiments, estimation of the parameters is generally based on ordinary least squares (OLS). However, in the presence of multicollinearity and outliers, OLS can result in very poor estimates. In this case, effects due to the combined outlier-multicollinearity problem can be reduced to certain extent by using alternative approaches. One of these approaches is to use biased-robust regression techniques for the estimation of parameters. In this paper, we evaluate various ridge-type robust estimators in the cases where there are multicollinearity and outliers during the analysis of mixture experiments. Also, for selection of biasing parameter, we use fraction of design space plots for evaluating the effect of the ridge-type robust estimators with respect to the scaled mean squared error of prediction. The suggested graphical approach is illustrated on Hald cement data set.

## 1. Introduction

Mixture experiments, a special study area of response surface methodology (RSM), are performed in many areas of product development and improvement. The purpose of mixture experiments is to model the blending surface with some forms of the mathematical equation. In general, for modeling well-behaved mixture system, the specific models such as Scheffé canonical polynomials are used in developing the appropriate response model. The parameter estimates of the Scheffé canonical polynomials are obtained by the ordinary least squares (OLS) method. However, in mixture experiments, multicollinearity is more frequent than the other experimental studies because of the constraints of components composing the mixture. Therefore, OLS estimates of the Scheffé canonical polynomials may be statistically insignificant with wrong sign and their variances are large so they may be far from the true values due to constraints on the mixture components [1–3]. To reduce the effect of multicollinearity in the analysis of mixture experiments, variable transformation or ordinary ridge regression (ORR), suggested by Hoerl and Kennard [4], is widely used as a classical method.

In mixture experiments, the detailed application of ORR estimator is given by St. John [1]. To evaluate the performance of ORR estimator, the graphical approaches apart from the analytical methods have been proposed in mixture experiments. These graphical approaches are based on plots of the scaled prediction variance (SPV) trace, which is suggested by Vining et al. [5]. The plot of SPV traces is used to compare the quality of prediction for designs in mixture experiments [5, 6]. Using the plot of SPV traces, Jang and Yoon [7] suggested a graphical method for evaluating and selecting an appropriate biasing parameter *k* that balances the bias-variance tradeoff. With this approach, the SPV values are plotted along prediction rays that correspond to Cox directions. On the other hand, Jang and Anderson-Cook [8] used fraction of design space (FDS) plots, which is developed by Zahran et al. [9], and violin plots to illustrate and evaluate the effect of ORR estimator with respect to the SPV values and to guide the decision about the value of biasing parameter *k*.

In mixture experiments, apart from the problems due to multicollinearity, another important problem which needs attention in parameter estimation is the presence of outliers in the data set [3, 10, 11]. In this case, outliers affect the efficiency of OLS estimates [12]. To overcome this problem, robust estimation methods like *M*-estimator, least median of squares (LMS) estimator, least trimmed sum of squares (LTS) estimator, *S*-estimator, and MM-estimator are proposed (see [12–16]). However, although robust estimators are the resistant techniques used to obtain the parameter estimates which are not affected by outliers, they are frequently unstable when the design matrix is ill-conditioned [12]. Therefore, effects due to both outliers and multicollinearity in the analysis of mixture experiments can be reduced to a certain extent by using alternative approaches. One of these approaches is to use biased-robust estimators for the estimation of the coefficients. In literature, it is recommended to use alternative ridge-type robust estimators for both outliers and multicollinearity problem in data set (see [17–22]).

The purpose of this study is to apply and evaluate various ridge-type robust estimators in the analysis of mixture experiments. Also, the performance of ridge-type robust estimators is dependent on the biasing parameter. In this case, to determine the biasing parameter apart from the analytical methods, alternative plots which are used in comparison and evaluation of the designs and which are based on the SPV values or the scaled mean squared error of prediction (SMSEP) values can be used. Considering the SPV values in evaluating and comparing designs is important, but bias properties of designs should also be considered [23]. Therefore, our study will focus on the use of FDS plots as a graphical tool for evaluating the effect of various ridge-type robust estimators with respect to the SMSEP values and for guiding the decision about the value of the biasing parameter.

This paper is organized as follows. In the next section, the concise information about the analysis of mixture experiments with robust methods is given. In Section 3, the ridge-type robust estimators based on the *M*-estimator, GM-estimator, and MM-estimator are introduced. In Section 4, the FDS plots are introduced for the selection of biasing parameter *k*. The suggested approach is illustrated on Hald cement data in Section 5. Finally, the obtained results are given in Section 6.

## 2. Mixture Experiments

In mixture experiments, the measured response depends only on the proportions of the components present in the mixture and not on the amount of the mixture. For example, the measured response might be the strength of concrete which is a mixture of cement, sand, and water, or it might be octane rating of a blend of gasoline. The aim of these experiments is to find optimal component proportions that provide desired values of some product performance characteristics [24].

Let *x*_*i*_ represent the proportion of the *i*th component present in a *q* component mixture; then

(1) $$\begin{array}{c} 0\leq x_{i}\leq 1,\;\;i=1,2,\ldots ,q,\;\overset{q}{\underset{i=1}{\sum }}x_{i}=1. \end{array}$$

The mixture constraints (1) produce a regular (*q* − 1)-dimensional simplex experimental region. However, the experimental region (1) is sometimes subjected to additional constraints on individual mixture components of the form

(2) $$\begin{array}{c} 0\leq L_{i}\leq x_{i}\leq U_{i}\leq 1\;i=1,2,\ldots ,q, \end{array}$$

where *L*_*i*_ and *U*_*i*_ denote lower and upper bounds, respectively. In general, restriction (2) reduces the experimental region given by (1) to an irregular (*q* − 1)-dimensional hyperpolyhedron. Mixture experimental designs are discussed by Cornell [24] for regular and irregular simplex regions.

In general, the presence of natural constraints or/and some additional linear constraints between the components requires the use of specific polynomial models such as Scheffé canonical polynomials for the modeling of the mixture system [25]. For modeling well-behaved mixture system, generally the Scheffé canonical polynomials are sufficient [24]. The first- and second-degree Scheffé canonical polynomial are given as

(3) $$\begin{array}{c} y=\overset{q}{\underset{i=1}{\sum }}\beta_{i}x_{i}\\ y=\overset{q}{\underset{i=1}{\sum }}\beta_{i}x_{i}+\overset{q}{\underset{i=1}{\sum }}\overset{q}{\underset{i<j}{\sum }}\beta_{ij}x_{i}x_{j}. \end{array}$$

As usual, we can represent the Scheffé canonical polynomial models in matrix form by

(4) $$\begin{array}{c} y=X\beta +\varepsilon , \end{array}$$

where **y** is *n* × 1 vector of observations on the response variable, **X** is *n* × *p*(≥*q*) matrix, where *p* is number of the terms in the model, **β** is *p* × 1 vector of parameters to be estimated, and **ε** is *n* × 1 vector of random errors satisfying *E*(**ε**) = 0 and *V*(**ε**) = *σ* ^2^ **I**. The vector of unknown parameters **β** can be generally estimated using OLS method. The OLS estimator for **β** is

(5) $$\begin{array}{c} {\hat{\beta }}_{\text{OLS}}={\left(X^{\prime }X\right)}^{-1}X^{\prime }y. \end{array}$$

When the observations **y** in model (4) are normally distributed, the OLS is a good parameter estimation procedure in the sense that it produces an estimator of the parameter vector **β** that has good statistical properties [12]. For example, ${\hat{\beta }}_{\text{OLS}}$ is the best linear unbiased estimator of **β** and also the maximum-likelihood estimator of **β**. On the other hand, there are many situations where the distribution of the response variable is nonnormal. Under conditions of nonnormal distributions, particularly heavy-tailed distributions, OLS estimator no longer has these desirable properties. These heavy-tailed distributions tend to generate outliers, and these outliers may have improper effect on the OLS estimates. To deal with this, several robust-to-outliers methods have been proposed in the literature [12, 16].

Robust regression methods are well-designed statistical techniques to produce regression coefficients which are not sensitive to outliers and nonnormal error distributions [13]. The most popular of all robust estimation techniques is *M* estimation, proposed by Huber [26]. The *M*-estimator is defined as

(6) $$\begin{array}{c} {\hat{\beta }}_{M}=\underset{\beta }{\arg\;\min}\overset{n}{\underset{u=1}{\sum }}\rho \left(\frac{e_{u}\left(\beta \right)}{s}\right), \end{array}$$

where *e*_*u*_ denote the residuals as $e_{u}=e_{u}\left(\hat{\beta }\right)=y_{u}-x_{u}^{\prime }\hat{\beta }$, where **x**_*u*_′ denote the *u*th row of **X**, and *s* is a robust estimate of scale. The scale *s* is required to gain scale equivariance and either can be an external scale estimate or can be estimated simultaneously. The function *ρ* is related to the likelihood function for an appropriate choice of the error distribution. Different choices for *ρ* lead to different variants of *M*-estimators. As with most *M*-estimation procedures, both the Huber weight function and the Tukey biweight function are two common choices [16].

To minimize (6), equate the first partial derivatives of *ρ* with respect to each parameter which leads to *p* equations of the form

(7) $$\begin{array}{c} \overset{n}{\underset{u=1}{\sum }}x_{uj}\psi \left(\frac{e_{u}\left(\beta \right)}{s}\right)=0,\;j=1,2,\ldots ,p, \end{array}$$

where *ψ*(·) is the partial derivative of *ρ* with respect to parameters. In general, the *ψ* function in (7), a nonlinear function, must be solved by iterative methods. The most common solution is iteratively reweighted least squares (IRLS), resulting in an estimator

(8) $$\begin{array}{c} {\hat{\beta }}_{M}={\left(X^{\prime }WX\right)}^{-1}X^{\prime }Wy, \end{array}$$

where **W** is an *n* × *n* diagonal matrix of weights *w*_*u*_ = *ψ*(*e*_*u*_/*s*)/(*e*_*u*_/*s*), *u* = 1,2,…, *n*. IRLS estimation procedure updates **W** matrix until a convergence criterion has been met [12].

On the other hand, *M*-estimators are not robust to high leverage points. As a remedy to this problem, bounded-influence generalized *M*-estimators (GM-estimators) have been proposed. The most commonly used GM-estimator is the Schweppe-type estimator. Handschin et al. [27] proposed an objective function with associated normal equations of the form

(9) $$\begin{array}{c} \overset{n}{\underset{u=1}{\sum }}\psi \left(\frac{e_{u}\left(\beta \right)}{\pi_{u}s}\right)\pi_{u}x_{u}=0, \end{array}$$

where, for appropriate values of *π*_*u*_ = *π*_*u*_(**x**_*u*_), the GM-estimator can downweight outliers with high leverage points. Several suggestions for *π*_*u*_ have been made that involve typical OLS outlier diagnostics, including studentized residuals or DFFITS [28]. The resulting estimator is (8), except the weights which become *w*_*u*_ = *ψ*(*e*_*u*_/*π*_*u*_ *s*)/(*e*_*u*_/*π*_*u*_ *s*). In the literature, several GM estimation approaches are suggested using various combinations of GM components (objective function, initial estimate, scale estimate, *π* function, etc.) [29, 30].

The breakdown point and efficiency are two practical concerns that should be taken into account when selecting a robust estimation procedure [12, 16]. According to these properties, GM-estimators possess the same efficiency and asymptotic distributional properties as *M*-estimators. But their breakdown points may not be much better than the worst case 1/*n*. In these cases, both *M*-estimation and GM-estimation can be improved by starting with a good initial estimate. In general, an alternative desirable initial estimator with a high breakdown point can be *S*-estimator [29, 30].

*S*-estimator proposed by Rousseeuw and Yohai [14] is based on minimizing the dispersion of the residuals. The objective function for *S*-estimation is

(10) $$\begin{array}{c} {\hat{\beta }}_{S}=\underset{\beta }{\arg\;\min}{\hat{\sigma }}_{S}\left(e_{1}\left(\beta \right),\ldots ,e_{n}\left(\beta \right)\right), \end{array}$$

where the dispersion function ${\hat{\sigma }}_{S}$ is found implicitly as the solution to

(11) $$\begin{array}{c} \frac{1}{n}\overset{n}{\underset{u=1}{\sum }}\rho \left(\frac{e_{u}\left(\beta \right)}{{\hat{\sigma }}_{S}}\right)=\kappa , \end{array}$$

where *κ* is a tuning constant defined as *κ* = *E*_Φ_[*ρ*(*e*)] and Φ represent the standard normal distribution. The solution ${\hat{\sigma }}_{S}$ is called an *M*-estimator of scale. But it is impossible for *S*-estimator to achieve a high breakdown point as well as a high efficiency [16].

Other most commonly used robust estimators, proposed by Yohai [15], are MM-estimators, which combine high breakdown with high asymptotic efficiency. Estimates are a modification of the typical *M*-estimates and are obtained through a three-step process [16]. Let ${\hat{\beta }}_{S}$ be an *S*-estimator, and let ${\hat{\sigma }}_{S}$ be the corresponding *M*-estimator of scale, solving (11) for $\beta ={\hat{\beta }}_{S}$. The MM-estimator is then defined as local solution of

(12) $$\begin{array}{c} {\hat{\beta }}_{\text{MM}}=\underset{\beta }{\arg\;\min}\overset{n}{\underset{u=1}{\sum }}\rho \left(\frac{e_{u}\left(\beta \right)}{{\hat{\sigma }}_{S}}\right) \end{array}$$

obtained with IRLS and initial value ${\hat{\beta }}_{S}$. The initial *S*-estimator guarantees a high breakdown point and the final MM-estimate a high Gaussian efficiency. In both the initial *S*-estimator and the final MM-estimator, a standard choice of *ρ* is Tukey biweight function. The tuning constant *κ* can be set to 1.547 for the *S*-estimator to guarantee a 50% breakdown point, and it can be set to 4.685 for the second step MM-estimator in (12) to guarantee a 95% efficiency of the final estimator [16].

## 3. Ridge-Type Robust Estimators

Multicollinearity and outliers are two main problems for researchers using regression estimation methods. In the presence of multicollinearity, several alternative estimation techniques are proposed. One of them is the ORR estimator which was proposed by Hoerl and Kennard [4]. The ORR estimator which is the most popular method for dealing with multicollinearity is defined by

(13) $$\begin{array}{c} {\hat{\beta }}_{\text{ORR}}={\left(X^{\prime }X+kI\right)}^{-1}X^{\prime }X{\hat{\beta }}_{\text{OLS}},\;k>0, \end{array}$$

where *k* is called biasing parameter. Since ${\hat{\beta }}_{\text{ORR}}$ depends on ${\hat{\beta }}_{\text{OLS}}$, ${\hat{\beta }}_{\text{ORR}}$ is sensitive to outliers. Therefore, outliers may have also improperly effect on the ridge estimates. The problem is further complicated when both outliers and multicollinearity are present in the data [31].

In recent years, major efforts have been made to obtain reliable estimates especially in the presence of outliers and also multicollinearity. When both outliers and multicollinearity occur in a data set, it would seem beneficial to combine methods designed to deal with these problems individually. It is hoped that the resulting ridge-type robust estimators will be resistant to outliers and less affected by multicollinearity [19, 21, 31].

A ridge-type *M*-estimator, suggested by Silvapulle [19], is defined by

(14) $$\begin{array}{c} {\hat{\beta }}_{\text{RM}}={\left(X^{\prime }X+kI\right)}^{-1}X^{\prime }X{\hat{\beta }}_{M}=Z{\hat{\beta }}_{M},\;k>0, \end{array}$$

where ${\hat{\beta }}_{M}$ is the *M*-estimators of **β**. Silvapulle [19] shows that $\text{MSE}\left[{\hat{\beta }}_{\text{RM}}\right]<\text{MSE}\left[{\hat{\beta }}_{\text{ORR}}\right]$ is obtained under some conditions and a positive *k*_1_ exists which depends on the unknown parameters such that $\text{MSE}\left[{\hat{\beta }}_{\text{RM}}\right]<\text{MSE}\left[{\hat{\beta }}_{M}\right]$, for 0 < *k* < *k*_1_.

On the other hand, Maronna [22] stated that **Z** matrix and ${\hat{\beta }}_{M}$ estimator in (14) are also sensitive to leverage points. Therefore, a ridge-type robust estimator which is not sensitive to both *x*- and *y*-outliers is given by

(15) $$\begin{array}{c} {\hat{\beta }}_{\text{RR}}={\left(X^{\prime }WX+kI\right)}^{-1}X^{\prime }WX{\hat{\beta }}_{R},\;k>0, \end{array}$$

where ${\hat{\beta }}_{R}$ is one of the robust estimators (such as GM-estimator or MM-estimator) of **β**. In this case, the use of ridge-type robust estimators is a good method for obtaining more stable parameter estimates for the model.

The ridge-type robust estimator given in (15) depends on the biasing parameter *k*. For selection of biasing parameter, Silvapulle [19] proposed the two different types of adaptive choices based on Hoerl et al. [32] (abbreviated to HKB) and Lawless and Wang [33] (abbreviated to LW). The same results can be extended to ${\hat{\beta }}_{\text{RR}}$ estimators. The resulting two choices of *k* are

(16) $$\begin{array}{c} {\hat{k}}_{\text{HKB}}=\frac{p{\hat{A}}^{2}}{{||{\hat{\beta }}_{R}||}^{2}},\;\;{\hat{k}}_{\text{LW}}=\frac{p{\hat{A}}^{2}}{\sum_{i=1}^{p}\lambda_{i}\beta_{Ri}^{2}}, \end{array}$$

where ${\hat{A}}^{2}=s^{2}\left\{{\left(n-p\right)}^{-1}\sum \psi^{2}\left(e_{u}/s\right)\right\}{\left\{n^{-1}\sum \psi^{\prime }\left(e_{u}/s\right)\right\}}^{-2}$ and *λ*_*j*_ is the *j*th eigenvalue of **X**′**W** **X**. Also for ORR estimator, there are several methods for selecting the biasing parameter *k* in literature. Most of these methods have been reviewed by Clark and Troskie [34]. Recommended methods can be similarly extended to the ridge-type robust estimators.

## 4. The Graphical Method for Evaluating Biasing Parameter

Due to the fact that there is not a definite rule for selection of biasing parameter, the graphical approaches developed for the ORR estimator can be used to select the biasing parameter of the ridge-type robust estimators. Different than analytical methods, determination of the biasing parameter with graphical approach is useful since it helps the researcher to examine the prediction properties of design based on the different biasing parameters visually. The prediction properties of a design, specifically the SMSEP values, also depend on the fitted model. Therefore multicollinearity due to the fitted model also influences the performance of the prediction properties of design completely. In this case, to examine the graphical approaches developed for comparison of the performance of designs based on the different biasing parameter for a single design is a useful method [7, 8].

For using the suggested methods on the analysis of mixture experiments, firstly we define the predicted response at **x**_*u*_. When we use ${\hat{\beta }}_{\text{RR}}$ estimator, the predicted response value at **x**_*u*_ is given as

(17) $$\begin{array}{c} {\hat{y}}_{k}\left(x_{u}\right)=x_{u}^{\prime }{\hat{\beta }}_{\text{RR}}=x_{u}^{\prime }{\left(X^{\prime }WX+kI\right)}^{-1}X^{\prime }WX{\hat{\beta }}_{R}. \end{array}$$

The mean squared error of ${\hat{y}}_{k}\left(x_{u}\right)$, also known as the mean squared error of prediction (MSEP), is given by

(18) $$\begin{array}{c} \text{MSE}\left[{\hat{y}}_{k}\left(x_{u}\right)\right]=\text{var}\left[{\hat{y}}_{k}\left(x_{u}\right)\right]+{\left\{\text{Bias}\left[{\hat{y}}_{k}\left(x_{u}\right)\right]\right\}}^{2}, \end{array}$$

where $\text{Bias}\left[{\hat{y}}_{k}\left(x_{u}\right)\right]=E\left[{\hat{y}}_{k}\left(x_{u}\right)\right]-y\left(x_{u}\right)$ is the bias associated with estimating $\hat{y}\left(x_{u}\right)$.

The variance of ${\hat{y}}_{k}\left(x_{u}\right)$, also known as the prediction variance, is given by

(19) var[y^k(xu)]=xu′(X′WX+kI)−1X′WXvar[β^R] ×X′WX(X′WX+kI)−1xu,

where $\text{var}\left[{\hat{\beta }}_{R}\right]$ is the variance-covariance matrix of ${\hat{\beta }}_{R}$. There are various approximations for determination of covariance matrix of the robust estimators in the literature [13]. Huber [13] has shown that a reasonable approximation for the covariance matrix of ${\hat{\beta }}_{R}$ is $\text{var}\left[{\hat{\beta }}_{R}\right]\approx K^{2}{\hat{A}}^{2}{\left(X^{\prime }X\right)}^{-1}$, where *K* = 1 + (*p*/*n*)(var(*ψ*′(*e*_*u*_))/[*E*(*ψ*′(*e*_*u*_))]^2^) is correction factor.

Using (17), the bias of ${\hat{y}}_{k}\left(x_{u}\right)$ at **x**_*u*_ is approximately given by

(20) $$\begin{array}{c} \text{Bias}\left[{\hat{y}}_{u}\left(x_{u}\right)\right]\approx x_{u}^{\prime }\left[{\left(X^{\prime }WX+kI\right)}^{-1}X^{\prime }WX-I\right]\beta . \end{array}$$

By combining the result in (19) and (20), an approximate expression for the MSE of ${\hat{y}}_{k}\left(x_{u}\right)$ is given by

(21) MSE[y^k(xu)]≈K2A2xu′(X′WX+kI)−1X′WX(X′X)−1 ×X′WX(X′WX+kI)−1xu+k2xu′ ×(X′WX+kI)−1ββ′(X′WX+kI)−1xu.

Note that $\text{MSE}\left[{\hat{y}}_{k}\left(x_{u}\right)\right]$ is a function of the biasing parameter *k*, the location of mixture **x**_*u*_ in the design space, the model matrix, and the weights matrix. It should also be noted that $\text{MSE}\left[{\hat{y}}_{k}\left(x_{u}\right)\right]$ depends on **β**, which is unknown. To calculate the $\text{MSE}\left[{\hat{y}}_{k}\left(x_{u}\right)\right]$ in (21), the unknown parameters can be replaced by suitable estimates of the parameters.

When design comparison focuses on prediction, the MSEP is generally scaled by *n*/*σ* ^2^ to create a scale-free quantity that reflects the design size. The scaled mean squared error of prediction (SMSEP) at the *u*th observation for a ridge-type robust estimator is written as

(22) $$\begin{array}{c} \text{SMSE}\left[{\hat{y}}_{k}\left(x_{u}\right)\right]=\frac{n}{s^{2}}\text{MSE}\left[{\hat{y}}_{k}\left(x_{u}\right)\right], \end{array}$$

where *n* denotes the design size. The multiplication by *n* serves to adjust the MSE for the total number of observations. This practice, which is quite common in RSM, makes it possible to use the SMSEP as a design criterion for comparing several designs [6].

In an experimental region, determination of the mixture points **x**_*u*_, $\text{SMSE}\left[{\hat{y}}_{k}\left(x_{u}\right)\right]$ values along with a constant *n* value can be compared for different *k* values. Instead of comparing $\text{SMSE}\left[{\hat{y}}_{k}\left(x_{u}\right)\right]$ values arbitrarily, a graphical approach which examines the distribution of the values of $\text{SMSE}\left[{\hat{y}}_{k}\left(x_{u}\right)\right]$ on the experimental region can be used. So, an appropriate *k* > 0 value which achieves $\text{SMSE}\left[{\hat{y}}_{k}\left(x_{u}\right)\right]<\text{SMSE}\left[\hat{y}\left(x_{u}\right)\right]$ condition on the experimental region can be determined.

To evaluate the distribution of $\text{SMSE}\left[{\hat{y}}_{k}\left(x_{u}\right)\right]$ values on the constrained experimental design, the FDS plots which are used in comparison and evaluation of the designs can be used. The main advantage of the use of FDS plots is to display characteristics of $\text{SMSE}\left[{\hat{y}}_{k}\left(x_{u}\right)\right]$ values throughout a multidimensional design region on a two-dimensional graph. Therefore, we can use the FDS plots as a graphical tool for evaluating the effect of ridge-type robust estimators with respect to the SMSEP values and for selecting an appropriate value of the biasing parameter *k*. In this case, instead of just considering a single FDS curve for a design, we examine the impact of the biasing parameter on the MSEP performance throughout the experimental region.

To calculate the FDS, two sampling methods with nearly same results are generally explored to sample mixture points **x**_*u*_ from the design space [35]. The first uses shrunken regions by Piepel et al. [36] and randomly selects a number of points on each shrinkage level proportional to that level. The second method selects points completely at random that fit within the constraints of the region [35]. In this study, we will use second method for generation of the sample points on the experimental region. Once the points are generated, the $\text{SMSE}\left[{\hat{y}}_{k}\left(x_{u}\right)\right]$ values are calculated for each point. The empirical cumulative distribution function is then calculated for the $\text{SMSE}\left[{\hat{y}}_{k}\left(x_{u}\right)\right]$ values and plotted on the FDS plot. The minimum $\text{SMSE}\left[{\hat{y}}_{k}\left(x_{u}\right)\right]$ value is shown at FDS of 0 and the maximum value is shown at the fraction 1. Along the FDS curve, the fraction of the design space at or below a particular $\text{SMSE}\left[{\hat{y}}_{k}\left(x_{u}\right)\right]$ value can be determined. A good design starts with small values and remains flat throughout. Therefore, with FDS plot, we see the relationship between the different biasing parameters *k* and we see the pattern of $\text{SMSE}\left[{\hat{y}}_{k}\left(x_{u}\right)\right]$ changing as *k* increases. By examining several different values of *k*, we can determine the smallest possible value of *k*, which stabilizes the $\text{SMSE}\left[{\hat{y}}_{k}\left(x_{u}\right)\right]$ distribution.

## 5. Example: The Hald Cement Data

To evaluate the performances of ridge-type robust estimators, we will draw on a mixture experiment given by Piepel and Redgate [37]. Piepel and Redgate [37] pointed out that the data from a cement example published by Hald [38] can be analyzed in the framework of the mixture experiments. The purpose of the experiment is to investigate the influence of the components on the heat of hardening (calories/gram), measured after the cements cured for 180 days. The data given in Table 1 contains the *x*_*i*_ values (weight fractions, wf) computed from the 13 oxide weight percent clinker compositions. Additional information on the analyzing of the Hald cement experiment can be found in Piepel and Redgate [37] and Smith [3].

When we use first-degree Scheffé canonical polynomial, the obtained model is as follows:

(23) $$\begin{array}{c} \hat{y}=\underset{\left(33.64\right)}{-433.7}x_{1}+\underset{\left(64.35\right)}{54.9}x_{2}-\underset{\left(32.21\right)}{252.7}x_{3}+\underset{\left(88.58\right)}{56.7}x_{4}+\underset{\left(16.76\right)}{309.2}x_{5}, \end{array}$$

where the quantities in parentheses are estimated as standard errors of the coefficient estimates. Statistics for detecting influential observations for the Hald cement data set are given in Table 2.

Based on the result of Table 2, *e*_8_ ordinary residual seems suspiciously large. Also, observation 3 has a high leverage value. The parameter estimates of the robust estimators are given in Table 3. In Table 3, the quantities in parentheses are estimated as standard errors of the coefficient estimates and AICR is the robust version of Akaike Information Criterion which is used for model selection [16].

Table 4 displays the final weights for robust estimators. In *M*-estimation, there is considerably downweight only at point 3. In case of GM-estimation, both point 3 and point 8 are significantly downweight. On the other hand, all observations are moderately downweight in MM-estimation. But, we note that neither point 3 nor point 8 is significantly downweight in MM-estimation, despite the fact that these points have the largest Cook's distance and ordinary residual, respectively.

In addition, the condition number of **X**′**X** matrix is 101.034. So, the design matrix **X** is moderately ill-conditioned. Therefore, due to the multicollinearity and outliers, the ridge-type GM-estimator or the ridge-type MM-estimator can be used. For the ridge-type GM-estimator, we can calculate the biasing parameter as ${\hat{k}}_{\text{HKB}}\approx {\hat{k}}_{\text{LW}}=0.00001$. For the ridge-type MM-estimator, the biasing parameter can be estimated as ${\hat{k}}_{\text{HKB}}=0.00007$ and ${\hat{k}}_{\text{LW}}=0.00002$ based on the methods proposed by Silvapulle [19].

**Figure 1:**
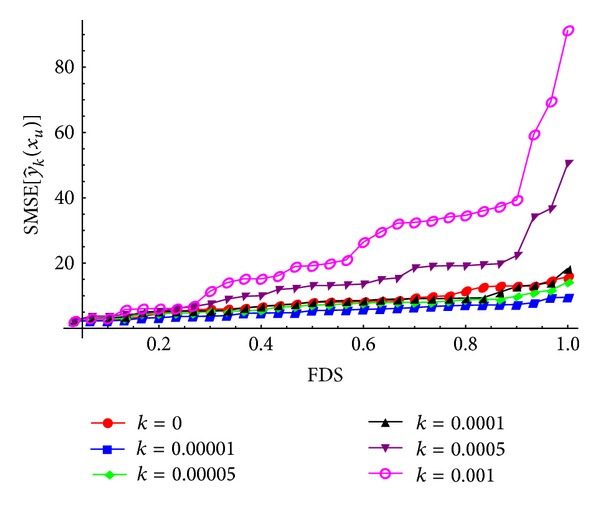
The FDS plots for different *k* values according to the randomly generated mixture, where ${{\hat{\beta }}_{R}=\hat{\beta }}_{\text{GM}}$.

**Figure 2:**
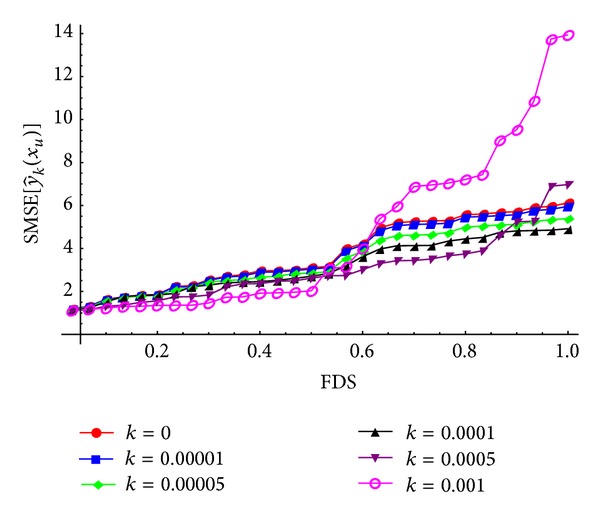
The FDS plots for different *k* values according to the randomly generated mixture, where ${{\hat{\beta }}_{R}=\hat{\beta }}_{\text{MM}}$.

To evaluate the biasing parameter *k* for the ridge-type GM-estimator and the ridge-type MM-estimator, randomly generated mixture points on the experimental region are considered. For different *k* values, the FDS plots of $\text{SMSE}\left[{\hat{y}}_{k}\left(x_{u}\right)\right]$ values based on ${\hat{\beta }}_{\text{GM}}$ and ${\hat{\beta }}_{\text{MM}}$ estimators are given in Figures 1 and 2, respectively.

In Figures 1 and 2, as the biasing parameter *k* increases, the distribution of $\text{SMSE}\left[{\hat{y}}_{k}\left(x_{u}\right)\right]$ values initially decreases gradually and then increases dramatically. In this case, we can select a reasonably positive small value of *k* at which the distribution of $\text{SMSE}\left[{\hat{y}}_{k}\left(x_{u}\right)\right]$ is stable and $\text{SMSE}\left[{\hat{y}}_{k}\left(x_{u}\right)\right]<\text{SMSE}\left[\hat{y}\left(x_{u}\right)\right]$ condition is achieved. For the ridge-type GM-estimator, the biasing parameter *k* is approximately chosen as *k* = 0.00001. Also, for the ridge-type MM-estimator, we can select the biasing parameter *k* as *k* = 0.0001. The obtained models along with $\text{MSE}\left[{\hat{y}}_{k}\left(x_{u}\right)\right]$ values are given in Table 5.

**Figure 3:**
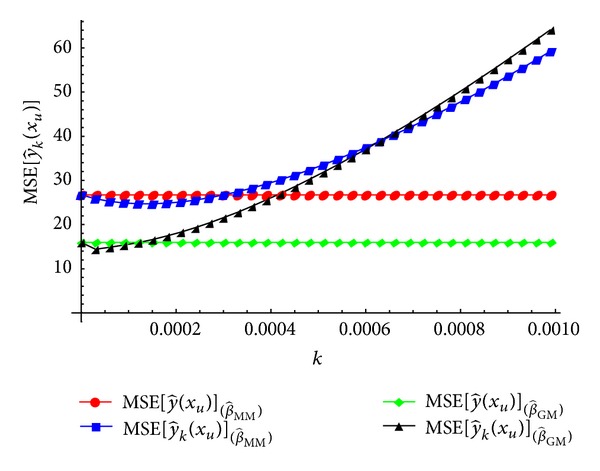
Comparison of the ridge-type GM-estimator and the ridge-type MM-estimator under MSEP sense.

In Tables 3 and 5, we observe that $\text{MSE}\left[{\hat{y}}_{k=0.00001}\left(x_{u}\right)\right]<\text{MSE}\left[\hat{y}\left(x_{u}\right)\right]$ is achieved for the ridge-type GM-estimator. Also, when we select the biasing parameter *k* as *k* = 0.0001, the same result is obtained for the ridge-type MM-estimator. A more comprehensive comparison of the ridge-type GM-estimator and the ridge-type MM-estimator under MSEP sense is given in Figure 3.

From Figure 3, it can be seen that $\text{MSE}{\left[{\hat{y}}_{k}\left(x_{u}\right)\right]}_{\left({\hat{\beta }}_{\text{GM}}\right)}$ is smaller than $\text{MSE}{\left[\hat{y}\left(x_{u}\right)\right]}_{\left({\hat{\beta }}_{\text{GM}}\right)}$ for *k* < 0.00012. Also, $\text{MSE}{\left[{\hat{y}}_{k}\left(x_{u}\right)\right]}_{\left({\hat{\beta }}_{\text{MM}}\right)}$ is smaller than $\text{MSE}{\left[\hat{y}\left(x_{u}\right)\right]}_{\left({\hat{\beta }}_{\text{MM}}\right)}$ for *k* < 0.00029. In addition, when we select the biasing parameter *k* as *k* < 0.0006, $\text{MSE}{\left[{\hat{y}}_{k}\left(x_{u}\right)\right]}_{\left({\hat{\beta }}_{\text{GM}}\right)}$ is smaller than $\text{MSE}{\left[{\hat{y}}_{k}\left(x_{u}\right)\right]}_{\left({\hat{\beta }}_{\text{MM}}\right)}$. For the values of biasing parameter *k* larger than *k* > 0.0006, $\text{MSE}{\left[{\hat{y}}_{k}\left(x_{u}\right)\right]}_{\left({\hat{\beta }}_{\text{MM}}\right)}$ is smaller than $\text{MSE}{\left[{\hat{y}}_{k}\left(x_{u}\right)\right]}_{\left({\hat{\beta }}_{\text{GM}}\right)}$. As a result, which ridge-type robust-estimator is the best seems to be dependent on the robust estimator of **β** and the choice of biasing parameter *k*. We have to replace these unknown parameters by some suitable estimates in practice. Therefore, the results of the graphical findings may change.

## 6. Conclusions

In this study, we used various ridge-type robust estimators to reduce the effect of the multicollinearity and outliers on the model parameters to certain extent in the analysis of mixture experiments. We observed that the performance of the ridge-type robust estimator depends on the robust estimator of **β** and the biasing parameter *k*. In this study, as robust estimator of **β**, the *M*-estimator, GM-estimator, and MM-estimator were discussed. By selecting the robust estimators which are less sensitive to points outlying in the *x*-and/or *y*-direction will improve the performance of the ridge-type robust-estimator. On the other hand, depending on the biasing parameter, to evaluate the performance of the ridge-type robust estimators, we adapted extensive enlargement of the graphical approach suggested for ORR estimator. For evaluating the biasing parameter, the FDS plots which are based on the SMSEP values are considered. As known in literature, the MSEP is a more powerful criterion for comparison of the designs than the prediction variance. Because MSEP incorporates the prediction variance and the prediction bias associated with the fitted model, the results obtained by FDS plots which are based on the SMSEP values were observed to be consistent with the other analytic approaches used in literature. Therefore, for evaluation of biasing parameter, the FDS plots which are based on the SMSEP values can also be used for both the mixture models affected by multicollinearity and outliers and other biased-robust estimators.

## Figures and Tables

**Table 1:** Hald cement data.

ID	*x*_1_ SiO_2_ (wf)	*x*_2_ Al_2_O_3_ (wf)	*x*_3_ Fe_2_O_3_ (wf)	*x*_4_ MgO (wf)	*x*_5_ CaO (wf)	*y* H_180_ (cal/g)
1	0.2742	0.0376	0.0198	0.0248	0.6436	78.5
2	0.2600	0.0350	0.0510	0.0230	0.6310	74.3
3	0.2181	0.0568	0.0279	0.0498	0.6474	104.3
4	0.2465	0.0581	0.0281	0.0240	0.6433	87.6
5	0.2500	0.0390	0.0210	0.0240	0.6660	95.9
6	0.2226	0.0619	0.0279	0.0239	0.6637	109.2
7	0.2098	0.0462	0.0572	0.0211	0.6657	102.7
8	0.2357	0.0481	0.0722	0.0221	0.6219	72.5
9	0.2220	0.0464	0.0616	0.0232	0.6468	93.1
10	0.2129	0.0876	0.0119	0.0249	0.6627	115.9
11	0.2252	0.0501	0.0751	0.0220	0.6276	83.8
12	0.2132	0.0611	0.0290	0.0260	0.6707	113.3
13	0.2183	0.0558	0.0269	0.0239	0.6751	109.4

**Table 2:** Statistics for detecting influential observations for the Hald cement data.

ID	*y*	$\hat{y}$	*e*_*i*_	*r*_*i*_	*e*_(*i*)_	*h*_*ii*_	Cook's Distance	DFITS
1	78.5	78.532	−0.032	−0.020	−0.019	0.489	0.000	−0.018
2	74.3	72.703	1.596	0.856	0.840	0.286	0.059	0.533
3	104.3	104.495	−0.195	−0.863	−0.848	0.989	14.074	−8.240
4	87.6	89.487	−1.887	−0.979	−0.976	0.236	0.059	−0.543
5	95.9	95.721	0.178	0.101	0.095	0.362	0.001	0.071
6	109.2	106.403	2.796	1.370	1.465	0.144	0.063	0.603
7	102.7	104.108	−1.408	−0.835	−0.817	0.415	0.099	−0.689
8	72.5	75.722	−3.222	−1.845	−2.277	0.373	0.406	−1.758
9	93.1	92.049	1.050	0.530	0.505	0.195	0.013	0.249
10	115.9	115.777	0.122	0.101	0.094	0.699	0.004	0.144
11	83.8	81.430	2.369	1.342	1.426	0.359	0.202	1.069
12	113.3	112.417	0.882	0.446	0.422	0.197	0.009	0.209
13	109.4	111.649	−2.249	−1.176	−1.210	0.249	0.092	−0.697

**Table 3:** The parameter estimates of the robust estimators.

${\hat{\beta }}_{R}$	${\hat{\beta }}_{1}$	${\hat{\beta }}_{2}$	${\hat{\beta }}_{3}$	${\hat{\beta }}_{4}$	${\hat{\beta }}_{5}$	$\hat{s}$	AICR	$\text{MSE}\left[\hat{y}\left(x_{u}\right)\right]$
${\hat{\beta }}_{M}$	$\underset{\left(35.823\right)}{-431.244}$	$\underset{\left(68.522\right)}{59.539}$	$\underset{\left(34.294\right)}{-248.274}$	$\underset{\left(94.323\right)}{57.195}$	$\underset{\left(17.845\right)}{307.774}$	2.118	15.196	27.599
${\hat{\beta }}_{\text{GM}}$	$\underset{\left(27.238\right)}{-444.933}$	$\underset{\left(52.10\right)}{44.524}$	$\underset{\left(26.075\right)}{-222.962}$	$\underset{\left(71.717\right)}{677.678}$	$\underset{\left(13.568\right)}{290.069}$	1.335	72.534	15.956
${\hat{\beta }}_{\text{MM}}$	$\underset{\left(35.199\right)}{-432.122}$	$\underset{\left(67.328\right)}{56.987}$	$\underset{\left(33.697\right)}{-250.218}$	$\underset{\left(92.680\right)}{57.286}$	$\underset{\left(17.534\right)}{308.365}$	2.519	10.304	26.690

**Table 4:** Final weights from robust regressions.

ID	*M*-estimate	GM-estimate	MM-estimate
1	1	1	0.9999
2	1	1	0.9669
3	1	0.0015	0.9994
4	1	1	0.9476
5	1	1	0.9993
6	1	1	0.8896
7	1	1	0.9715
8	0.8268	0.4595	0.8456
9	1	1	0.9856
10	1	1	0.9997
11	1	1	0.9281
12	1	1	0.9879
13	1	1	0.9314

**Table 5:** The parameter estimates of the ridge-type robust estimators.

${\hat{\beta }}_{\text{RR}}$	${\hat{\beta }}_{1}$	${\hat{\beta }}_{2}$	${\hat{\beta }}_{3}$	${\hat{\beta }}_{4}$	${\hat{\beta }}_{5}$	$\text{MSE}{\left[{\hat{y}}_{k}\left(x_{u}\right)\right]}_{\left({\hat{\beta }}_{\text{RR}}\right)}$
${\hat{\beta }}_{\text{RR}}{\left({\hat{\beta }}_{\text{GM}}\right)}_{\left(k=0.00001\right)}$	$\underset{\left(27.121\right)}{-431.634}$	$\underset{\left(51.491\right)}{62.523}$	$\underset{\left(25.808\right)}{-230.555}$	$\underset{\left(32.155\right)}{304.331}$	$\underset{\left(13.274\right)}{297.865}$	14.124
${\hat{\beta }}_{\text{RR}}{\left({\hat{\beta }}_{\text{MM}}\right)}_{\left(k=0.0001\right)}$	$\underset{\left(35.011\right)}{-419.691}$	$\underset{\left(66.697\right)}{72.054}$	$\underset{\left(33.578\right)}{-243.282}$	$\underset{\left(91.264\right)}{55.259}$	$\underset{\left(17.385\right)}{302.402}$	26.382
